# Air–breathing behavior underlies the cell death in limbs of *Rana pirica* tadpoles

**DOI:** 10.1186/s40851-022-00199-x

**Published:** 2023-01-09

**Authors:** Satomi F. Ono, Ingrid Rosenburg Cordeiro, Osamu Kishida, Haruki Ochi, Mikiko Tanaka

**Affiliations:** 1 School of Life Science and Technology, Tokyo Institute of Technology, B–17, 4259 Nagatsuta–cho, Midori–ku, Yokohama, Kanagawa 226–8501 Japan; 2 Tomakomai Experimental Forest, Field Science Center for Northern Biosphere, Hokkaido University, Tomakomai, Hokkaido 053–0035 Japan; 3 Institute for Promotion of Medical Science Research, Faculty of Medicine, Yamagata University, 2–2–2 Iida–Nishi, Yamagata, Yamagata 990–9585 Japan

**Keywords:** Limb development, Interdigital cell death, Reactive oxygen species, Evolution

## Abstract

Amphibians shape their limbs by differential outgrowth of digits and interdigital regions. In contrast, amniotes employ cell death, an additional developmental system, to determine the final shape of limbs. Previous work has shown that high oxygen availability is correlated with the induction of cell death in developing limbs. Given the diversity of life histories of amphibians, it is conceivable that some amphibians are exposed to a high–oxygen environment during the tadpole phase and exhibit cell death in their limbs. Here, we examined whether air–breathing behavior underlies the cell death in limbs of aquatic tadpoles of the frog species *Rana pirica*. Our experimental approach revealed that *R. pirica* tadpoles exhibit cell death in their limbs that is likely to be induced by oxidative stress associated with their frequent air–breathing behavior.

**Supplementary Information:**

The online version contains supplementary material available at 10.1186/s40851-022-00199-x.

## Background

The shapes of the hands and feet are crucial for tetrapods to adapt to their habitats. In amphibians, the balance between the growth of the digits and interdigital regions determines whether the hands and feet are webbed [1, 2]. However, in amniotes, the interdigital regions are actively removed by cell death to determine the final shape of the limbs [3–5]. Cell death has enabled the evolution of a great variety of limb morphologies in amniotes, including the webbing in ducks, bats, and turtles [6–8], and the lack of toes in horses and camels [9]. Understanding how this novel developmental mechanism appeared in amniotes is important for elucidating the mechanisms underlying evolution of limb morphology.

Despite the importance of cell death in the interdigital regions, the molecular mechanisms promoting it remain largely unclear. Bmp signaling is one of the critical pathways required for cell death to shape amniote limbs [10–13]. Recent studies have revealed an additional factor involved in this process. The production of reactive oxygen species (ROS) is key for interdigital cell death [14–16]. Changes in environmental oxygen levels alter the numbers of dying cells in the interdigital regions of chickens and mice in a ROS–dependent manner [14, 16, 17], indicating that environmental oxygen directly controls cell death in developing limbs.

It has long been appreciated that cell death does not occur in the interdigital regions of amphibians [1, 2]. However, the corresponding studies examined amphibians with biphasic life histories including an aquatic tadpole phase. Unlike early reports [1, 2], later studies described cell death in the interdigital regions in two species, the seepage salamanders (*Desmongnathus aeneus*) [18] and the coqui frogs (*Eleutherodactylus coqui*) [17]. These amphibians have terrestrial eggs and undergo direct–development on land. Surprisingly, increasing atmospheric oxygen tension or increasing interdigital blood vessel density is sufficient to induce ROS production and cell death in the interdigital regions of aquatic tadpoles of *Xenopus laevis*, suggesting that the interdigital regions of amphibians have the molecular background to induce cell death in response to increased levels of environmental oxygen which is supplied via interdigital blood vessels [17].

In amniote eggs, the highly vascularized extraembryonic membranes exchange oxygen with the air to provide an abundant oxygen supply to growing embryos. Thus, interdigital tissue of amniote embryos is supplied with sufficient levels of oxygen to produce ROS required for the induction of cell death [17]. However, amphibians do not have extraembryonic membranes; thus, larvae or tadpoles rely on the cutaneous respiration [19] as well as the development of respiratory organs, lungs and gills, for oxygen uptake [20]. Furthermore, aquatic habitats are often hypoxic; consequently, water–breathing using gills and cutaneous exchange can be insufficient for species dwelling in such environments [20]. Thus, the presence of lungs is important to enable air-breathing in several aquatic amphibian species [21]. The respiratory strategies have an adaptive significance and are deeply connected with ecological factors as the presence of predators as well as the tadpole’s feeding and locomotion strategies [20]. Therefore, in addition to direct-developing species, it is conceivable that some amphibians with biphasic life histories would be exposed to high levels of atmospheric oxygen during the hindlimb formation stage and exhibit cell death in developing limbs.

Here, we examined whether air-breathing behavior influences oxygen availability during the development of the amphibian *Rana pirica* and leads to the induction of cell death in developing limbs. *Rana pirica* is a typical frog species that develops through an aquatic tadpole stage into an adult, and its tadpoles seemed to rise to the water surface to breath relatively frequently (Fig. S3a, Movies 1 and 2). Our experimental approach revealed that limbs of *R. pirica* tadpoles exhibit cell death that is likely induced by oxidative stress associated with their frequent air–breathing behavior.

## Methods

### Animals

*Rana pirica* tadpoles were collected in Hokkaido, Japan, from June to September, kept in filtered water, and staged according to [22, 23]. We further subdivided the four- to five-digit formation stages (stages 36 to 37) into five stages (see Fig. S1 for details) to provide a more detailed view on limb development. African clawed frog (*Xenopus laevis*) tadpoles were kept in 0.1% sea–salt solution at 16 °C and staged according to a developmental table [24]. Post–metamorphic froglets of *R. pirica* and *X. laevis* were photographed with a single–lens reflex camera (D5300, Nikon) (Fig. S2).

### Lung morphology

For observation of lungs, *R. pirica* tadpoles at stages 31–37 were anesthetized with 0.003% tricaine methanesulfonate in water, and lungs were dissected immediately under a dissecting microscope to observe the presence of air bubbles as well as vascularization. For anatomical studies, lungs dissected from early stage 37 tadpoles were stained with hematoxylin and eosin as described [25] with slight modifications. Briefly, dissected lungs were fixed in 4% paraformaldehyde (PFA) overnight, and then dehydrated in a graded ethanol series. Ethanol was then replaced with acetone, and the specimens were embedded in Technovit 8100 resin (Heraues–Kulzer). Sections were cut at a thickness of 12 μm, stained with hematoxylin, washed in tap water, stained with eosin, washed in tap water, treated with ethanol, xylene and covered in Mount Quick (Daido Sangyo).

### Frequency of air–breathing

The frequency of air–breathing behavior of *X. laevis* and *R. pirica* tadpoles (Fig. S3a; Movies 1 and 2) was compared at stages 54–56 and 36–37, the stages in which all five digits of hindlimbs are formed, respectively. The same water source and conditions were used for both species. The number of times the mouth of each tadpole reached the water surface was determined during a total of 10 minutes, excluding times when the tadpole was resting for more than 1 minute. For each species, three individuals were used per experiment, and the experiments were conducted three times each. The air–breathing behavior of *R. pirica* tadpoles was photographed with a single–lens reflex camera (D5300, Nikon).

### Detection of cell death and ROS

Cell death was detected using LysoTracker, Nile Blue, or TUNEL, as previously described [17, 26, 27]. LysoTracker labels the increased lysosomal activity detected in dying cells and around phagocytosed cell debris [28, 29] and has been used to identify the cell death in limb buds of various tetrapods, including *X. laevis* and coqui frogs [17, 30]. Briefly, tadpoles at stages 32–37 were incubated with 0.5 μM LysoTracker Red (Thermo Fisher Scientific, Waltham, MA) in PBS+ (PBS pH 7.4, 9 mM CaCl_2_, 3.3 mM MgCl_2_) for 2 hours, washed and photographed with na LSM780 confocal microscope (Zeiss). Some of the LysoTracker Red-stained tadpoles were then stained with 0.01% Nile blue in filtered water for 20 minutes, washed, and photographed. For TUNEL staining, hindlimb buds from stage 36 tadpoles were cryosectioned at 8–12 μm as described [17], and stained using TUNEL Mix (In situ Cell Death Kit, Roche) according to the manufacturer’s protocol. For detection of cell death and reactive oxygen species, tadpoles at stage 36–37 were incubated with 0.5 μM LysoTracker Green and with 2 μM CellROX Deep Red (Invitrogen) in PBS+ for 2 hours, washed, and photographed with an LSM780 confocal microscope (Zeiss).

### Oxygen incubation

Oxygen incubation of tadpoles were carried out as described [17]. Briefly, incubations under high (60%) oxygen atmosphere were made using a multigas incubator (APM–30D, Astec). Normoxic (21%) controls were incubated at atmospheric oxygen levels using a regular incubator (FMU–053, Fukushima Industries). Oxygen levels in the water were measured using a dissolved oxygen meter (DO–5509, Satotech). Tadpoles were immersed in 1.8 cm depth filtered water at 25 °C individually in plastic plates for 3 hours. After oxygen incubation, tadpoles were stained with LysoTracker and photographed, and the number of LysoTracker-positive cells was counted.

### Cell counting

We counted the number of the LysoTracker-positive cells as described [31], with modifications. The interdigital region was defined as the area between the outlines of digit primordia recognized by the confocal image obtained through the T–PMT channel. Then, the confocal image was processed using Fiji, ImageJ (https://fiji.sc). Images were converted to 8–bit images, adjusted for contrast, nonlinearly transformed with the gamma function, and binarized with the threshold. We considered the particle size from 5 to 8 to 200 pixels as cells, which were counted using the Analyze Particles function.

### Visualization of blood vessels

Blood vessels were visualized as previously described [17, 31]. Briefly, yellow highlighter ink diluted 1:1 in PBS (PUSR80.2 cartridge, Mitsubishi Pencil) was injected using pulled glass capillaries (Narishige) into the blood vessels of anesthetized tadpoles at stages 36 and 37. After 10 minutes, the samples were fixed with 4% PFA and photographed.

### Manipulation of air breathing behavior

For short term incubation, *R. pirica* tadpoles at early stage 36 were reared overnight in a 500 ml beaker without a plastic net (control), or with a plastic net to prevent the tadpoles from rising to the water surface (Fig. 3a). Tap water was filtered and poured into each beaker. For long-term incubation, *R. pirica* tadpoles at early stage 36 were reared freely (control) or in a 15 ml plastic tube covered with a plastic net for 3 days in a 1.5 L tank (Fig. 3b). Water in the 1.5 L tank was changed daily. The use of a plastic net stopped air breathing behavior, but did not interfere with the amount of dissolved oxygen in the beaker. After incubation in a beaker or a tank, tadpoles were stained with LysoTracker, photographed, and the number of LysoTracker-positive cells was counted.

### Statistical analysis

Statistical analyses were performed with Prism 8.0.2 (GraphPad). The data are presented as the mean ± SEM. Significance was determined using two–tailed unpaired *t*–tests for comparisons between different limbs. Significance was defined as *P* ≤ 0.05 (∗*p* < 0.05).

### Images

**Fig. 1:**
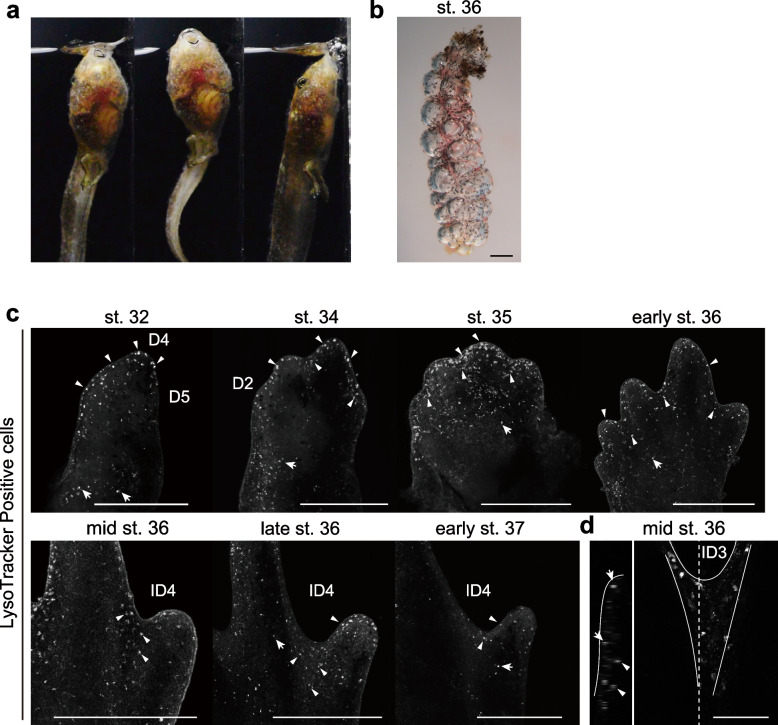
Air-breathing behavior, and cell death during limb development in *R. pirica*. **a** Air–breathing behavior of *R. pirica* tadpoles. **b** A lung dissected from a stage 36 *R. pirica* tadpole. **c** Cell death (LysoTracker Red) in *R. pirica* hindlimbs. LysoTracker–positive cells were found in interdigital regions, and limb margin edges (arrowheads), and surface ectoderm (arrows). **d** Cell death (LysoTracker Red) in mid stage 36 *R. pirica* hindlimbs. Cell death was observed in both the ectoderm (arrows) and the mesenchyme (arrowheads), as revealed on the orthogonal (optical) section of a confocal stack. D2, 4–5: digits 2, 4, and 5; ID3, 4: interdigital regions 3 and 4. Scale bars, 500 μm in (**b**), (**c**) and 100 μm in (**d**)

**Fig. 2:**
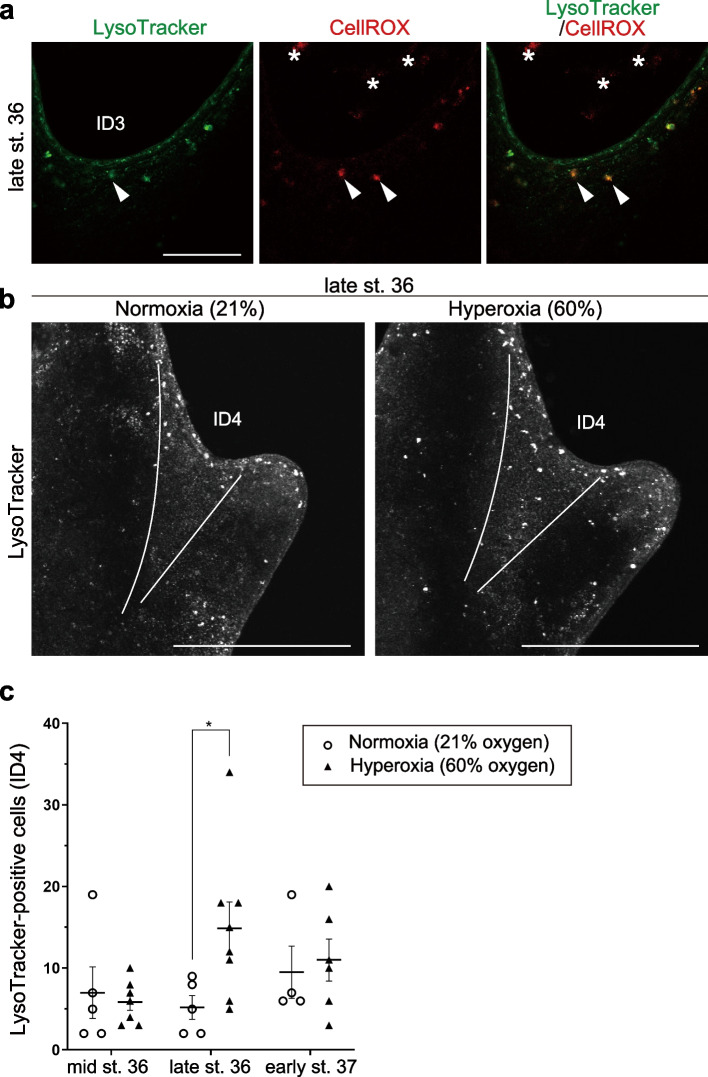
Distribution of cell death and ROS during limb development and experimental conditions in *R. pirica*. **a** Cell death (LysoTracker Green) and ROS (CellROX) staining of late stage 36 *R. pirica* hindlimbs. The arrowheads point to cells positive for both LysoTracker and CellROX staining. The asterisks are microbes, not stained cells. **b** Cell death (LysoTracker Red) in late stage 36 *R. pirica* hindlimbs incubated for 3 hours under normoxia or hyperoxia. **c** Quantification of LysoTracker–positive cells in ID4 of the hindlimbs indicated in (**b**). Each symbol represents an individual specimen. Mean ± SEM. Two–tailed unpaired *t*–test. ∗*p* < 0.05. ID3–4: interdigital regions 3 and 4. Scale bar, 100 μm in (**a**) and 500 μm in (**b**)

Images were captured using an LSM780 confocal microscope (Zeiss), stereomicroscope (MF16F, Leica; BX61WI, Olympus) with microscope digital camera (DP74, Olympus), or single–lens reflex camera (D5300, Nikon). Pseudocolor images were generated using Zen Black software (Zeiss). The following panels are maximum intensity projections of confocal image stacks: Figs. 1c, 2ab, S5a. The following panels were flipped horizontally: Fig. 1c (stage 32, stage 34, mid stage 36, and late stage 36), Fig. S5b.

## Results

### Air-breathing behavior, and cell death during limb development in *R. pirica* tadpoles

First, we examined the frequency of air–breathing behavior using the same water source for *X. laevis* and *R. pirica* tadpoles at stages 54–56 and 36–37, the stages in which all five digits of hindlimbs are formed, respectively (Fig. S3a, Movies 1 and 2). *Rana pirica* tadpoles rose to the surface nearly 10 times more often than *X. laevis* tadpoles (Fig. S3a), suggesting that *R. pirica* tadpoles obtain oxygen predominatly from air, compared with *X. laevis* tadpoles, at the examined stages under the same water condition.

Next, we examined whether pulmonary respiration occurs during hindlimb development stages of *R. pirica* tadpoles. The lungs of *X. laevis* tadpoles are inflated beginning at stage 46 (before hindlimb bud initiation) and are filled with air by stage 58 (when hindlimb morphogenesis is complete), the latter visualized by the presence of air bubbles [19, 32, 33]. Of the 11 lungs dissected from stage 31–35 *R. pirica* tadpoles, 45% contained air bubbles. At stage 36, of the 12 lungs dissected from tadpoles, 75% contained air bubbles (Fig. 1b). Of the six lungs dissected from tadpoles at stage 37, the stage in which there are five digits in hindlimbs, 100% contained air bubbles (Fig. S3b). In addition, we confirmed the presence of large blood vessels as well as the capillary vessels in the lungs in early stage 37 tadpoles (Fig. S3b, c; *n* = 3). These observations suggest that *R. pirica* tadpoles use their lungs for gas exchange at least by early stage 37.

Air–breathing behavior involving a rise to the surface may increase the efficiency of oxygen uptake in the tadpoles. The presence of cell death during limb development in chicken and mouse embryos, and experimentally in *X. laevis* tadpoles, is correlated with high oxygen availability [16, 17]. Thus, we examined whether cell death is induced in developing limb buds of *R. pirica* tadpoles by labelling them with LysoTracker (Fig. 1c, d). Surprisingly, we observed LysoTracker–positive cells in hindlimb buds of *R. pirica* tadpoles at all stages examined (Fig. 1c, stage 32, *n* = 1; stage 34, *n* = 1; stage 35, *n* = 2; early stage 36, *n* = 3; mid stage 36, *n* = 5; late stage 36, *n* = 9; early stage 37, *n* = 8). LysoTracker–positive cells were found preferentially in the interdigital regions, as well as in the limb margins (Figs. 1c, d; Fig. S4). We also detected some LysoTracker-positive cells in the surface ectoderm (Fig. 1c). In the interdigital regions, the LysoTracker–positive cells were observed in both the ectoderm and the mesenchyme (Figs. 1d; S4). We observed the same pattern using the exclusion dye Nile blue sulfate at stage 36 (Fig. S4 a; *n* = 6). When we used the TUNEL assay, a few apoptotic cells were detected in the limb margin (*n* = 6/6) and the interdigital region (*n* = 4/6) at stages 35–37 (Fig. S4c, d), as seen in the optical section image of LysoTracker-stained samples (Fig. S4b). These observations suggest that cell death occurs in developing limb buds of *R. pirica* tadpoles.

### Oxygen and ROS are correlated with cell death in limbs of *R. pirica* tadpoles

Cell death in limb buds is correlated with increased production of ROS, at least in mice, chickens, *Eleutherodactylus coqui*, and *X. laevis* [14, 15, 17]. Thus, we examined whether the cell death observed in *R. pirica* limbs is related to the production of ROS. In hindlimb buds of *R. pirica* tadpoles at mid stage 36 (*n* = 2), late stage 36 (*n* = 2), and early stage 37 (*n* = 2), high production of ROS was observed in LysoTracker–positive cells in both the limb margins and the interdigital regions (Figs. 2a and S5a), indicating that ROS levels are critical for the induction of the cell death in *R. pirica* limb buds.

In chickens and *X. laevis*, environmental oxygen levels directly affect ROS production within the limbs [17]. We thus tested whether increased atmospheric oxygen could promote cell death in the interdigital regions of *R. pirica*. Hyperoxia (60% oxygen) resulted in an approximately 1.7–fold increase in the amount of dissolved oxygen in the water (from 8.6 ± 0.8 ppm, SD, *n* = 5 to 14.7 ± 1.0 ppm, SD, *n* = 5). After 3 hours of incubation, the number of LysoTracker–positive cells was increased in the interdigital regions at late stage 36, but not at other stages under hyperoxia (Fig. 2b, c). These results suggest that high environmental oxygen directly promotes cell death in the hindlimbs of *R. pirica* tadpoles.

In addition to environmental oxygen, blood vessels are also essential for tissue oxygenation. Vascular networks provide oxygen and promote the production of ROS in the interdigital regions of mice and *X. laevis* [16, 17]. To visualize the vascular pattern, fluorescent ink was injected into blood vessels in *R. pirica* tadpoles. Dense networks of blood vessels were observed in the interdigital regions, as well as in the margins and the ectoderm (Fig. S5b, stage 36, *n* = 7; Fig. S5c, stage 37, *n* = 3), consistent with the distribution of LysoTracker–positive cells (Fig. 1c, d). Thus, a high blood vessel density seemed to provide an abundant source of oxygen in *R. pirica* hindlimb buds, which was correlated with ROS production and the occurrence of cell death in this species.

Taken together, the findings suggest that local oxygen availability in interdigital tissue is crucial for the induction of cell death in *R. pirica* hindlimbs.

### The air–breathing behavior of *R. pirica* tadpoles is related to the interdigital cell death

**Fig. 3:**
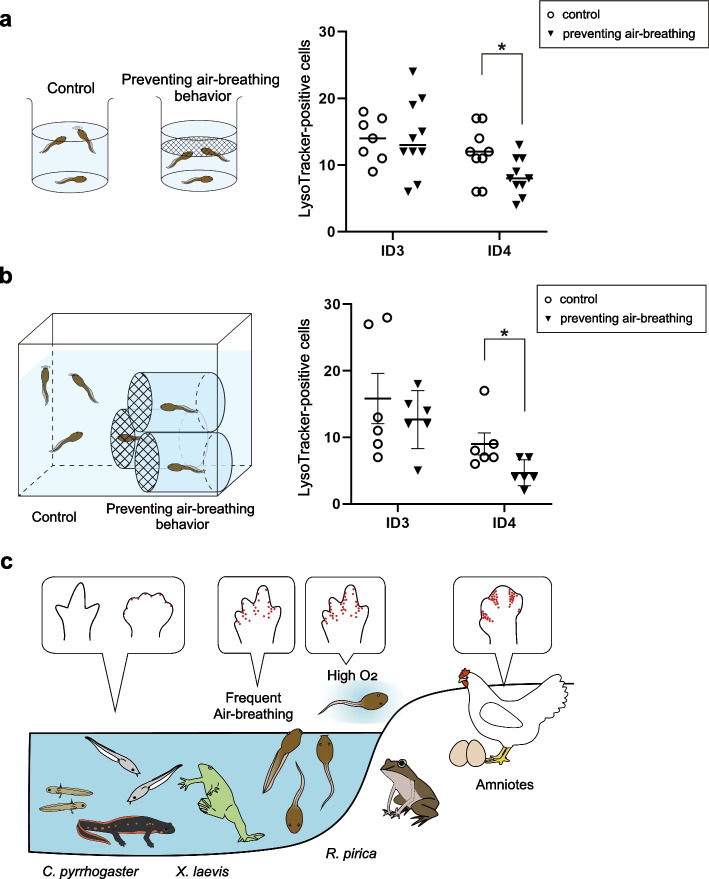
Air- breathing is correlated with cell death in *R. pirica* limbs. **a**, **b** Quantification of LysoTracker–positive cells in ID3, or ID4 of the hindlimbs of early stage 36 *R. pirica*, reared overnight in a container without a net or with a net (**a**) or for 3 days in a tank without or with a tube covered with a net (**b**). Each symbol represents an individual specimen. Mean + SEM. Two–tailed unpaired *t*–test. ∗p < 0.05. **c** Schematic illustrating the life history and limb development strategies of various amphibians. *X. laevis* and *C. pyrrhogaster* have aquatic tadpoles and no interdigital cell death [1, 17], while amniotes exhibit extensive interdigital cell death. Here we show that *R. pirica* has aquatic tadpoles, and exhibits interdigital cell death. The cell death observed in *R. pirica* tadpoles has been experimentally correlated to their frequent air–breathing behavior. Illustrations are modified after [17]

In light of the above results, we investigated whether the interdigital cell death in *R. pirica* is related to their frequent air–breathing behavior during hindlimb development stages. For this, early stage 36 tadpoles were reared overnight or for 3 days in a container with or without a net/a tube covered with a net (Fig. 3a, b). The tadpoles in which air–breathing was prevented had fewer LysoTracker–positive cells in the interdigital regions of the hindlimbs than the controls (Fig. 3a, b). These results suggest that the air–breathing behavior of *R. pirica* promotes cell death in the interdigital regions.

## Discussion

Here, we propose that air–breathing in aquatic larvae of amphibians greatly influences oxygen availability and leads to increased ROS production and cell death even in the limbs of aquatic tadpoles (Fig. 3c). Unlike amniotes, amphibian larvae and tadpoles have no extraembryonic membranes and thus rely greatly on the development of respiratory organs or other vascularized organs for oxygenation. Oxygen availability is a critical resource in aquatic habitats and has driven several adaptations throughout the evolution of amphibians [20]. Because of the complex life histories of this clade, the environmental oxygen levels of each species mostly depend on its habitats during the embryonic, larval, and adult phases [20]. Aquatic habitats, including ponds and small lakes, can be hypoxic if they are overpopulated or polluted, and be hyperoxic if there is little water [34]. It is therefore understandable that amphibians have developed stress–responsive systems to a highly oxygenated environment, including a system to remove cells damaged by oxidative stress. For aquatic larvae, it is also critical to survive under hypoxic conditions; thus, they can respond plastically to an oxygen-deficient environment by altering the use of various respiratory organs [20, 32]. Thus, it is likely that the prevention of the air-breathing behavior of *R. pirica* tadpoles in these experiments (Fig. 3a, b) led them to use respiratory organs other than the lungs, such as the gills or skins for oxygenation. However, this compensation was likely insufficient; and thus, oxygen–dependent cell death was reduced in limbs (Fig. 3a, b). Oxygenation from the air is an efficient method of oxygen uptake, but it also risks exposure to high oxidative stress.

In this study, LysoTracker-positive cells were observed in limb buds of *R. pirica* tadpoles at the earliest stage examined (Fig. 1c). As *R. pirica* tadpoles at this stage already surface to breathe air, they are exposed to high levels of atmospheric oxygen. In *R. pirica* limbs, LysoTracker-positive cells exhibited increased ROS production (Fig. 2a, Fig. S5a) and were found in the vicinity of the vascular network (Fig. S5b, c). Thus, in exchange for efficient oxygenation, some cells seemed to have been damaged by ROS derived from oxygen supplied via blood vessels, and such cells were likely removed by cell death. However, it is not clear whether the cell death observed in *R. pirica* plays some roles in shaping the limbs or is only a byproduct of increased ROS production. In fact, ectopic cell death induced in the interdigital regions of *X. laevis* under hyperoxic conditions contributes little to the final morphology [17]. We propose that the removal of damaged cells exposed to high levels of oxygen during development first appeared only as a byproduct of increased ROS production and that this mechanism could have been a first step of the evolutionary process toward the establishment of the cell death system. Future studies should reveal the process by which this new step was integrated into limb development and evolved into essential mechanisms to pattern amniote limbs.

Multiple integrations and reconstructions of pathways likely occurred during the evolution of the cell death system essential for limb patterning in amniotes. In addition to the production of ROS, the induction of cell death also requires the activities of various factors in the interdigital regions, such as Bmp signaling [10–13]. In addition to its role in cell death, interdigital Bmp signaling plays pivotal roles in periodic patterning of digits and interdigital regions [35, 36] as well as establishment of digit identity [37–39]. Interdigital Bmp signaling is active in limbs of the coqui frog [40], the axolotl [41], and *Xenopus laevis* [17], and its role in periodic patterning has been demonstrated even in the fins of chondrichthyans [42]. Thus, periodic patterning is likely at least a part of the ancestral role for Bmp signaling, which would have been established prior to the evolution of interdigital cell death [17, 43]. Likewise, the interdigital vascular networks, which are the sources of oxygen for interdigital tissue, play a fundamental role in skeletal morphogenesis, that is, in endochondral ossification of fingers [44, 45]. It would be fascinating if this process emerged in the vicinity of the vascular networks under a high-oxygen environment, was consequently integrated into the interdigital Bmp pathways regulating digital patterning, and evolved toward the essential developmental system. Investigation of the evolution of the roles of Bmp and ROS not only in amniotes but also in amphibians with various life histories will be required to fully elucidate the evolutionary process of cell death in amniote limbs.

## Conclusions

We propose that increased oxygen availability caused by air-breathing behavior leads to the production of ROS and the induction of cell death in developing limbs. An ecological shift may have led to unexpected morphological innovation that was integrated into developmental processes during evolution.

## Supplementary Information

**Additional file 1: Fig. S1.** Extended developmental stages of *R. pirica.***a** Stage 32 to 37 *R. pirica* tadpoles were identified by examination of hindlimbs according to the methods of [21] with slight modifications as done for *Hoplobatrachus rugulosus* [22]. Briefly, at stage 32, indentations of the 4^th^ and 5^th^ toes became visible; at stage 33, indentations of the 3^rd^ and 4^th^ toes appeared; at stage 34, indentations between the 2^nd^ and 3^rd^ toes appeared; at stage 35, the hindlimb margins showed indentations between all five toes; at stage 36, the 3^rd^ to 5^th^ toes were separated; and at stage 37, all toes were separated. To accurately evaluate the progression of cell death in developing limb buds, we further subdivided stage 36 and stage 37. Based on the length of the fourth digit (indicated as “L” in (b)), the following stages were used: early stage 36 (L < 400 µm), mid stage 36 (400 µm < L < 500 µm), late stage 36 (500 µm < L < 700 µm), and early stage 37 (700 µm < L < 900 µm). Dashed lines indicate the outlines of hindlimbs. Anterior is to the left. Scale bars, 1 mm. **b** Schematic diagram of the *R. pirica* hindlimb with the length of the fourth digit (D4) defined as “L”. D1-5: digits 1-5; Dist.: distal; Post.: posterior.**Fig. S2.***X. laevis* and *R. pirica* frogs and their hindlimbs. Interdigital webbings were observed in hindlimbs of both *X. laevis* and *R. pirica* post-metamorphic froglets. **Fig. S3.** Air breathing and lung blood vessels. **a** Frequency of air-breathing behavior of *X. laevis* and *R. pirica* tadpoles. Each symbol represents an individual specimen. Mean ± SEM. Two-tailed unpaired t-test. ∗*p*< 0.05. **b** A lung dissected from a stage 37 *R. pirica* tadpole. The presence of a large blood vessel (arrowheads) and thin capillary vessels (an arrow) were recognized. Scale bars, 200 µm. **c** Frontal section through the lungs of stage 37 *R. pirica* lungs. **c’, c”** Higher magnifications of the rectangles in (c). Blood vessels were defined in the lung wall (arrows). Scale bars, 500 µm in (c) and 100 µm in (c', c''). **Fig. S4.** Cell death detection in *R. pirica* hindlimbs. **a, a’** A hindlimb of stage 36 *R. pirica* tadpoles stained with LysoTracker Red and Nile Blue sulfate simultaneously. Note that both LysoTracker- and Nile Blue-signals were recognized in the same cells (arrowheads). **b** An optical (confocal) section of stage 36 *R. pirica* hindlimbs stained with LysoTracker. A few LysoTracker positive cells were detected in the interdigital region and the ectoderm (arrowheads). **c, d** TUNEL staining of stage 36 *R. pirica* hindlimbs. TUNEL positive cells were detected in the ectoderm (arrowheads in (c)) and the interdigital region (arrowheads in (d)). Scale bars, 100 µm. D4: digit 4; ID3-4: interdigital region 3-4. **Fig. S5.** Cell death, ROS staining and blood vessel pattern. **a **LysoTracker Green and CellROX staining of *R. pirica* hindlimbs at mid stage 36 and early stage 37. The arrowheads point to both LysoTracker- and CellROX-positive cells. The asterisks indicate pigments, not stained cells. **b** Vasculature (injected with fluorescent ink) of *R. pirica* hindlimbs stage 36. Vessels were observed in interdigital regions, limb margin edges (arrowheads), and surface ectoderm (arrows). **c** Vasculature (injected with fluorescent ink) of *R. pirica* hindlimbs (stage 37). Scale bars, 100 µm. D3: digit 3; ID3: interdigital region 3.

**Additional file 2: Movie 1.** Air-breathing behavior of *X. laevis* tadpoles.

**Additional file 3: Movie 2.** Air-breathing behavior of *R. pirica* tadpoles.

## Data Availability

Further information and requests for resources and reagents should be directed to and will be fulfilled by the Lead Contact, Mikiko Tanaka (mitanaka@bio.titech.ac.jp).

## References

[1] Cameron JA, Fallon JF (1977). The absence of cell death during development of free digits in amphibians. Dev Biol.

[2] Vlaskalin T, Wong CJ, Tsilfidis C (2004). Growth and apoptosis during larval forelimb development and adult forelimb regeneration in the newt (*Notophthalmus viridescens*). Dev Genes Evol.

[3] Fallon JF, Cameron J (1977). Interdigital cell death during limb development of the turtle and lizard with an interpretation of evolutionary significance. J Embryol Exp Morphol.

[4] Salas-Vidal E, Valencia C, Covarrubias L (2001). Differential tissue growth and patterns of cell death in mouse limb autopod morphogenesis. Dev Dyn.

[5] Fernandez-Teran MA, Hinchliffe JR, Ros MA (2006). Birth and death of cells in limb development: a mapping study. Dev Dyn.

[6] Hurle JM, Colvee E (1982). Surface changes in the embryonic interdigital epithelium during the formation of the free digits: a comparative study in the chick and duck foot. J Embryol Exp Morphol..

[7] Weatherbee SD, Behringer RR, Rasweiler JJ, Niswander LA (2006). Interdigital webbing retention in bat wings illustrates genetic changes underlying amniote limb diversification. Proc Natl Acad Sci U S A.

[8] Cordeiro IR, Yu R, Tanaka M (2020). Regulation of the limb shape during the development of the Chinese softshell turtles. Evol Dev.

[9] Cooper KL, Sears KE, Uygur A, Maier J, Baczkowski KS, Brosnahan M (2014). Patterning and post–patterning modes of evolutionary digit loss in mammals. Nature..

[10] Zou H, Niswander L (1996). Requirement for BMP signaling in interdigital apoptosis and scale formation. Science..

[11] Yokouchi Y, Sakiyama J, Kameda T, Iba H, Suzuki A, Ueno N, Kuroiwa A (1996). BMP–2/−4 mediate programmed cell death in chicken limb buds. Development..

[12] Zuzarte-Luis V, Hurle JM (2005). Programmed cell death in the embryonic vertebrate limb. Semin Cell Dev Biol.

[13] Kaltcheva MM, Anderson MJ, Harfe BD, Lewandoski M (2016). BMPs are direct triggers of interdigital programmed cell death. Dev Biol.

[14] Salas-Vidal E, Lomeli H, Castro-Obregon S, Cuervo R, Escalante-Alcalde D, Covarrubias L (1998). Reactive oxygen species participate in the control of mouse embryonic cell death. Exp Cell Res.

[15] Schnabel D, Salas-Vidal E, Narvaez V, del Rayo Sanchez-Carbente M, Hernandez-Garcia D, Cuervo R, Covarrubias L (2006). Expression and regulation of antioxidant enzymes in the developing limb support a function of ROS in interdigital cell death. Dev Biol.

[16] Eshkar-Oren I, Krief S, Ferrara N, Elliott AM, Zelzer E (2015). Vascular patterning regulates interdigital cell death by a ROS–mediated mechanism. Development..

[17] Cordeiro IR, Kabashima K, Ochi H, Munakata K, Nishimori C, Laslo M (2019). Environmental oxygen exposure allows for the evolution of interdigital cell death in limb patterning. Dev Cell.

[18] Franssen RA, Marks S, Wake D, Shubin N (2005). Limb chondrogenesis of the seepage salamander, *Desmognathus aeneus* (amphibia: plethodontidae). J Morphol.

[19] Phillips JR, Hewes AE, Womack MC, Schwenk K. The mechanics of air breathing in African clawed frog tadpoles, *Xenopus laevis* (Anura: Pipidae). J Exp Biol. 2022;225:jeb243102. 10.1242/jeb.243102 35481476

[20] Wells KD (2007). The Ecology and Behavior of Amphibians.

[21] Schwenk K, Phillips JR (2020). Circumventing surface tension: tadpoles suck bubbles to breathe air. Proc R Soc B.

[22] Gosner KL (1960). A simplified table for staging anuran embryos and larvae. Herpetologica..

[23] Traijitt T, Kitana N, Khonsue W, Kitana J (2021). Chronological changes in the somatic development of *Hoplobatrachus rugulosus* (Wiegmann, 1834) (Anura: Dicroglossidae). Trop Nat Hist.

[24] Nieuwkoop PD, Faber J (1994). Normal Table of *Xenopus laevis* (Daudin).

[25] Okamoto E, Van Mai H, Ishimatsu A, Tanaka M (2018). Modification of pectoral fins occurs during the larva–to–juvenile transition in the mudskipper (Periophthalmus modestus). Zoological Lett.

[26] Suda N, Itoh T, Nakato R, Shirakawa D, Bando M, Katou Y (2014). Dimeric combinations of MafB, cFos and cJun control the apoptosis–survival balance in limb morphogenesis. Development..

[27] Sanz-Ezquerro JJ, Tickle C (2000). Autoregulation of Shh expression and Shh induction of cell death suggest a mechanism for modulating polarising activity during chick limb development. Development..

[28] Zuzarte-Luis V, Montero JA, Kawakami Y, Izpisua-Belmonte JC, Hurle JM (2007). Lysosomal cathepsins in embryonic programmed cell death. Dev Biol.

[29] Boya P, Kroemer G (2008). Lysosomal membrane permeabilization in cell death. Oncogene..

[30] Fogel JL, Thein TZ, Mariani FV. Use of LysoTracker to detect programmed cell death in embryos and differentiating embryonic stem cells. J Vis Exp. 2012;(68):4254. 10.3791/4254 PMC3490301 23092960

[31] Takase Y, Tadokoro R, Takahashi Y (2013). Low cost labeling with highlighter ink efficiently visualizes developing blood vessels in avian and mouse embryos. Develop Growth Differ.

[32] Rose CS, James B (2013). Plasticity of lung development in the amphibian, *Xenopus laevis*. Biol Open.

[33] Phillips JR, Hewes AE, Schwenk K. The mechanics of air breathing in gray tree frog tadpoles, *Hyla versicolor* (Anura: Hylidae). J Exp Biol. 2020;223:jeb219311. 10.1242/jeb.219311 32041808

[34] Warkentin KM (2007). Oxygen, gills, and embryo behavior: mechanisms of adaptive plasticity in hatching. Comp Biochem Physiol A.

[35] Raspopovic J, Marcon L, Russo L, Sharpe J, Modeling digits. (2014). Digit patterning is controlled by a Bmp–Sox9–Wnt Turing network modulated by morphogen gradients. Science..

[36] Hiscock TW, Tschopp P, Tabin CJ (2017). On the formation of digits and joints during limb development. Dev Cell.

[37] Suzuki T, Hasso SM, Fallon JF (2008). Unique SMAD1/5/8 activity at the phalanx–forming region determines digit identity. Proc Natl Acad Sci U S A.

[38] Montero JA, Lorda-Diez CI, Ganan Y, Macias D, Hurle JM (2008). Activin/TGFbeta and BMP crosstalk determines digit chondrogenesis. Dev Biol.

[39] Huang BL, Trofka A, Furusawa A, Norrie JL, Rabinowitz AH, Vokes SA (2016). An interdigit signalling centre instructs coordinate phalanx–joint formation governed by 5’Hoxd–Gli3 antagonism. Nat Commun.

[40] Gross JB, Kerney R, Hanken J, Tabin CJ (2011). Molecular anatomy of the developing limb in the coqui frog, *Eleutherodactylus coqui*. Evol Dev.

[41] Guimond JC, Levesque M, Michaud PL, Berdugo J, Finnson K, Philip A, Roy S (2010). BMP–2 functions independently of SHH signaling and triggers cell condensation and apoptosis in regenerating axolotl limbs. BMC Dev Biol.

[42] Onimaru K, Marcon L, Musy M, Tanaka M, Sharpe J (2016). The fin–to–limb transition as the re–organization of a Turing pattern. Nat Commun.

[43] Cordeiro IR, Tanaka M (2020). Environmental oxygen is a key modulator of development and evolution: From molecules to ecology: Oxygen–sensitive pathways pattern the developing organism, linking genetic and environmental components during the evolution of new traits. Bioessays..

[44] Gerber HP, Vu TH, Ryan AM, Kowalski J, Werb Z, Ferrara N (1999). VEGF couples hypertrophic cartilage remodeling, ossification and angiogenesis during endochondral bone formation. Nat Med.

[45] Zelzer E, McLean W, Ng YS, Fukai N, Reginato AM, Lovejoy S (2002). Skeletal defects in VEGF(120/120) mice reveal multiple roles for VEGF in skeletogenesis. Development..

