# Supplementary material for: Air–breathing behavior underlies the cell death in limbs of Rana pirica tadpoles
Source: Zoological Lett. 2023 Jan 9;9:2. doi: 10.1186/s40851-022-00199-x (PMC9830891; doi:10.1186/s40851-022-00199-x)
Supplement: Supplementary file 1 — Additional file 1: Fig. S1. Extended developmental stages of R. pirica.a Stage 32 to 37 R. pirica tadpoles were identified by examination of hindlimbs according to the methods of [21] with slight modifications as done for Hoplobatrachus rugulosus [22]. Briefly, at stage 32, indentations of the 4th and 5th toes became visible; at stage 33, indentations of the 3rd and 4th toes appeared; at stage 34, indentations between the 2nd and 3rd toes appeared; at stage 35, the hindlimb margins showed indentations between all five toes; at stage 36, the 3rd to 5th toes were separated; and at stage 37, all toes were separated. To accurately evaluate the progression of cell death in developing limb buds, we further subdivided stage 36 and stage 37. Based on the length of the fourth digit (indicated as “L” in (b)), the following stages were used: early stage 36 (L < 400 µm), mid stage 36 (400 µm < L < 500 µm), late stage 36 (500 µm < L < 700 µm), and early stage 37 (700 µm < L < 900 µm). Dashed lines indicate the outlines of hindlimbs. Anterior is to the left. Scale bars, 1 mm. b Schematic diagram of the R. pirica hindlimb with the length of the fourth digit (D4) defined as “L”. D1-5: digits 1-5; Dist.: distal; Post.: posterior.Fig. S2.X. laevis and R. pirica frogs and their hindlimbs. Interdigital webbings were observed in hindlimbs of both X. laevis and R. pirica post-metamorphic froglets. Fig. S3. Air breathing and lung blood vessels. a Frequency of air-breathing behavior of X. laevis and R. pirica tadpoles. Each symbol represents an individual specimen. Mean ± SEM. Two-tailed unpaired t-test. ∗p< 0.05. b A lung dissected from a stage 37 R. pirica tadpole. The presence of a large blood vessel (arrowheads) and thin capillary vessels (an arrow) were recognized. Scale bars, 200 µm. c Frontal section through the lungs of stage 37 R. pirica lungs. c’, c” Higher magnifications of the rectangles in (c). Blood vessels were defined in the lung wall (arrows). Scale bars, 500 µm in (c) and [file 40851_2022_199_MOESM1_ESM.pdf]

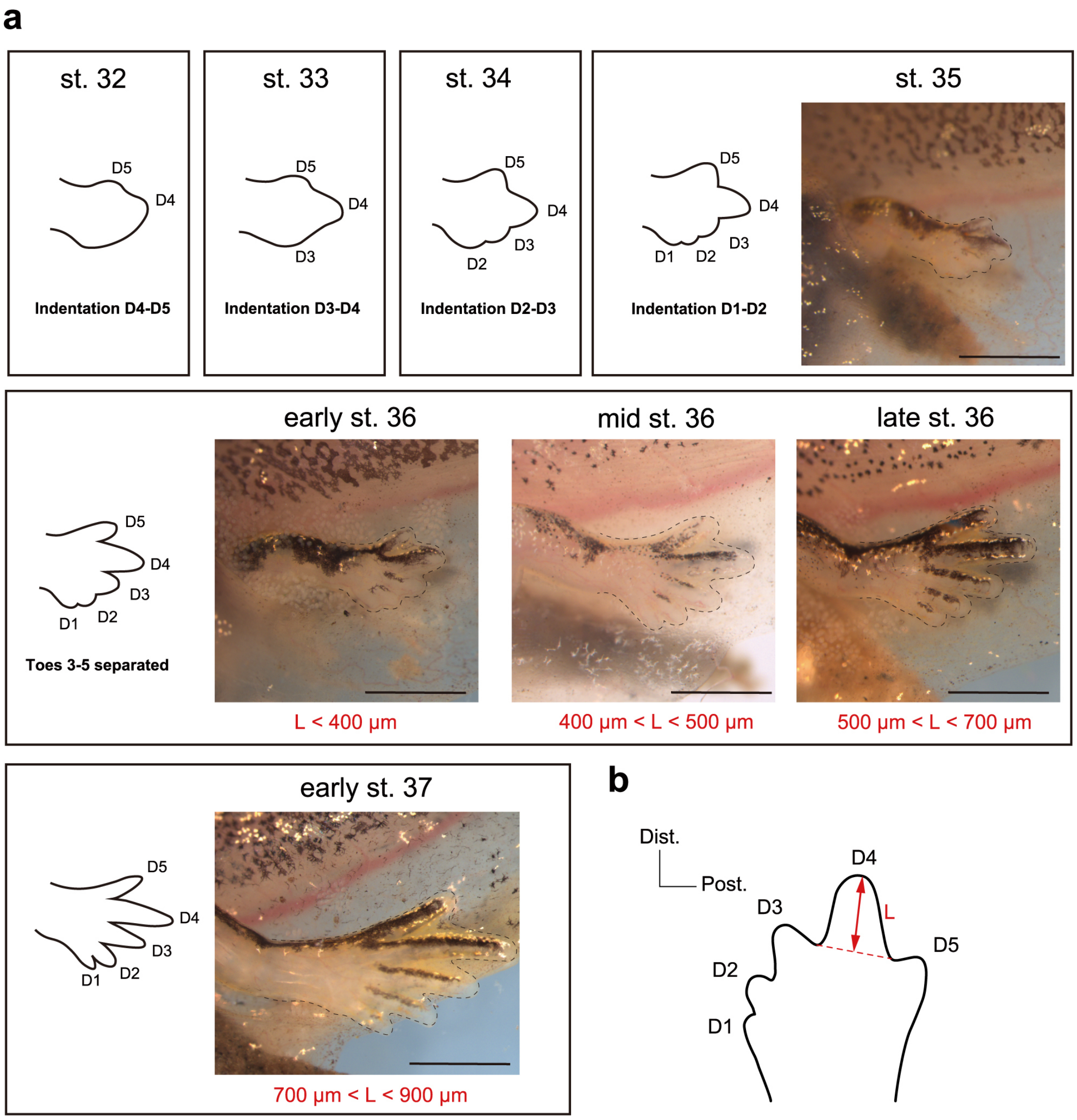

Fig. S1 Extended developmental stages of *R. pirica*

a Stage 32 to 37 *R. pirica* tadpoles were identified by examination of hindlimbs according to the methods of [21] with slight modifications as done for *Hoplobatrachus rugulosus* [22]. Briefly, at stage 32, indentations of the 4th and 5th toes became visible; at stage 33, indentations of the 3rd and 4th toes appeared; at stage 34, indentations between the 2nd and 3rd toes appeared; at stage 35, the hindlimb margins showed indentations between all five toes; at stage 36, the 3rd to 5th toes were separated; and at stage 37, all toes were separated. To accurately evaluate the progression of cell death in developing limb buds, we further subdivided stage 36 and stage 37. Based on the length of the fourth digit (indicated as "L" in (b)), the following stages were used: early stage 36 (L < 400 μm), mid stage 36 (400 μm < L < 500 μm), late stage 36 (500 μm < L < 700 μm), early stage 37 (700 μm < L < 900 μm). Dashed lines indicate the outlines of hindlimbs. Anterior is to the left. Scale bars, 1 mm. **b** Schematic diagram of the *R. pirica* hindlimb with the length of the fourth digit (D4) defined as "L". D1-5: digits 1-5; Dist.: distal; Post.: posterior.

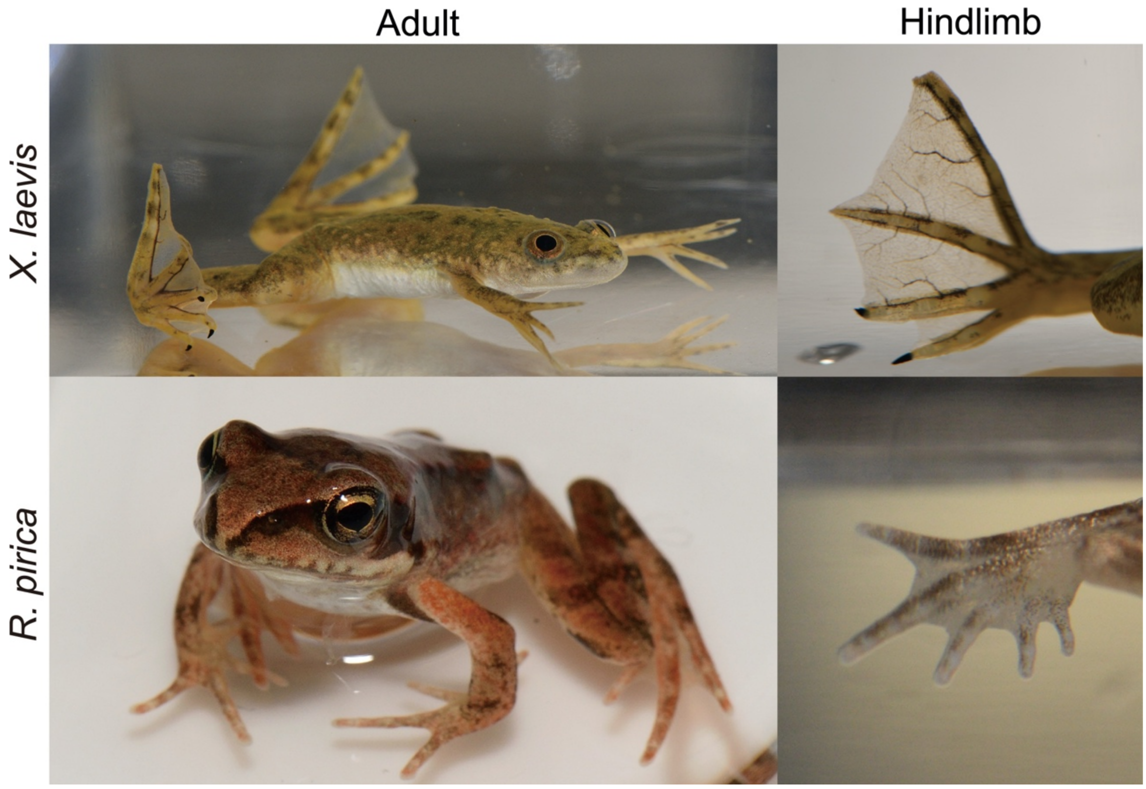

Fig. S2 *X. laevis* and *R. pirica* frogs and their hindlimbs

Interdigital webbings were observed in hindlimbs of both *X. laevis* and *R. pirica* post-metamorphic froglets.

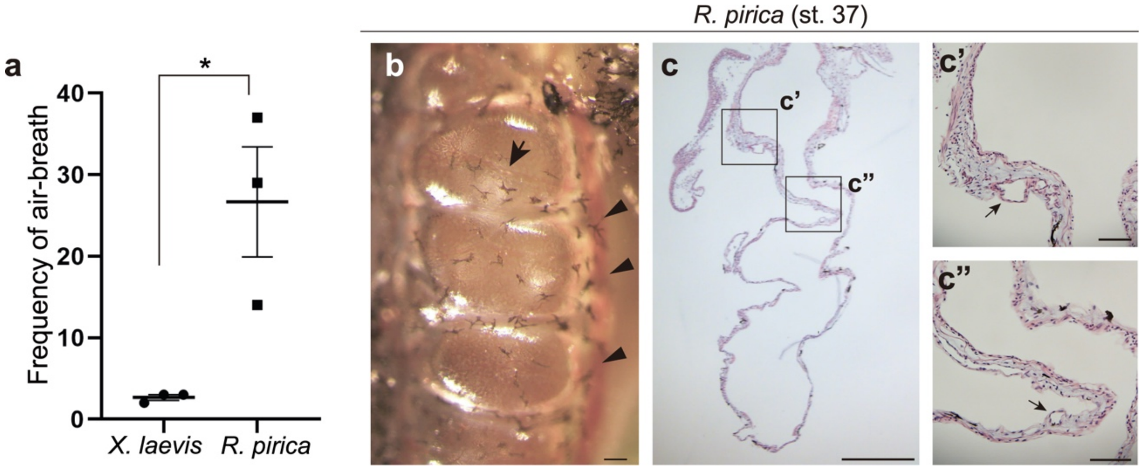

Fig. S3 Air breathing and lung blood vessels

a Frequency of air-breathing behavior of *X. laevis* and *R. pirica* tadpoles. Each symbol represents an individual specimen. Mean ± SEM. Two-tailed unpaired *t*-test. $*p < 0.05$. **b** A lung dissected from an stage 37 *R. pirica* tadpole. The presence of a large blood vessel (arrowheads) and thin capillary vessels (an arrow) were recognized. Scale bars, 200 µm. **c** Frontal section through the lungs of stage 37 *R. pirica* lungs. **c', c"** Higher magnifications of the rectangles in (c). Blood vessels were defined in the lung wall (arrows). Scale bars, 500 µm in (c) and 100 µm in (c', c").

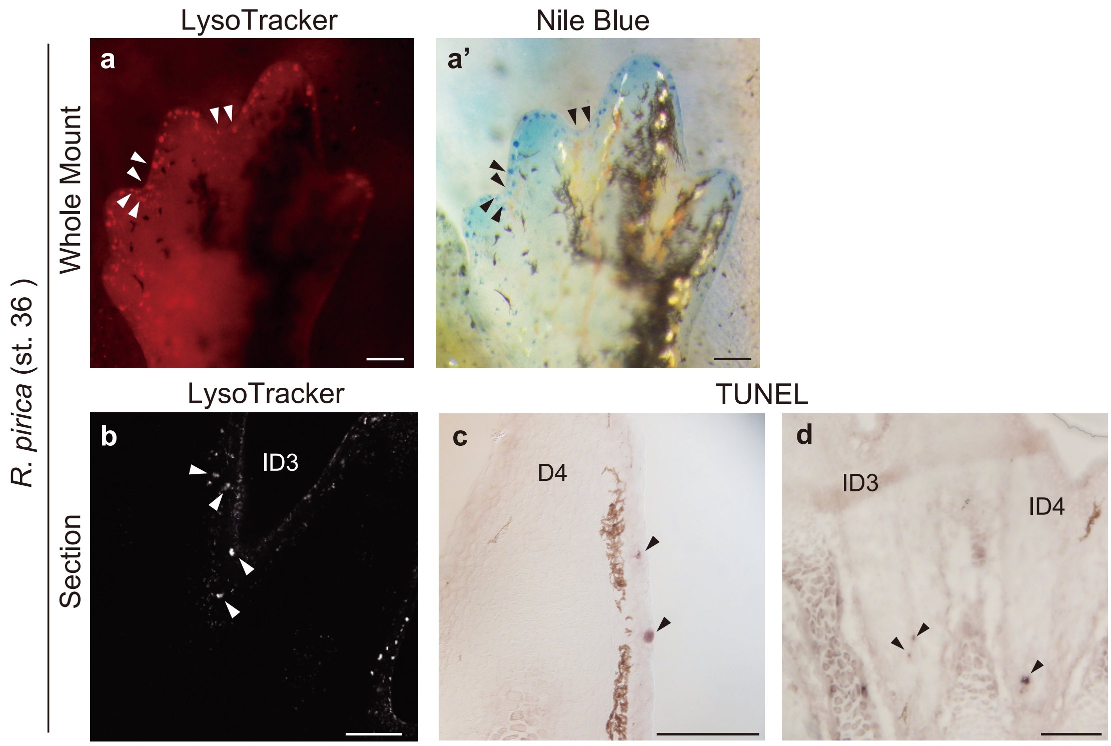

Fig. S4 Cell death detection in *R. pirica* hindlimbs

a, a' A hindlimb of stage 36 *R. pirica* tadpoles stained with LysoTracker Red and Nile Blue sulfate simultaneously. Note that both LysoTracker- and Nile Blue-signals were recognized in the same cells (arrowheads). **b** An optical (confocal) section of stage 36 *R. pirica* hindlimbs stained with LysoTracker. A few LysoTracker positive cells were detected in the interdigital region and the ectoderm (arrowheads). **c, d** TUNEL staining of stage 36 *R. pirica* hindlimbs. TUNEL positive cells were detected in the ectoderm (arrowheads in (c)) and the interdigital region (arrowheads in (d)). Scale bars, 100 μm. D4: digit 4; ID3-4: interdigital region 3-4.

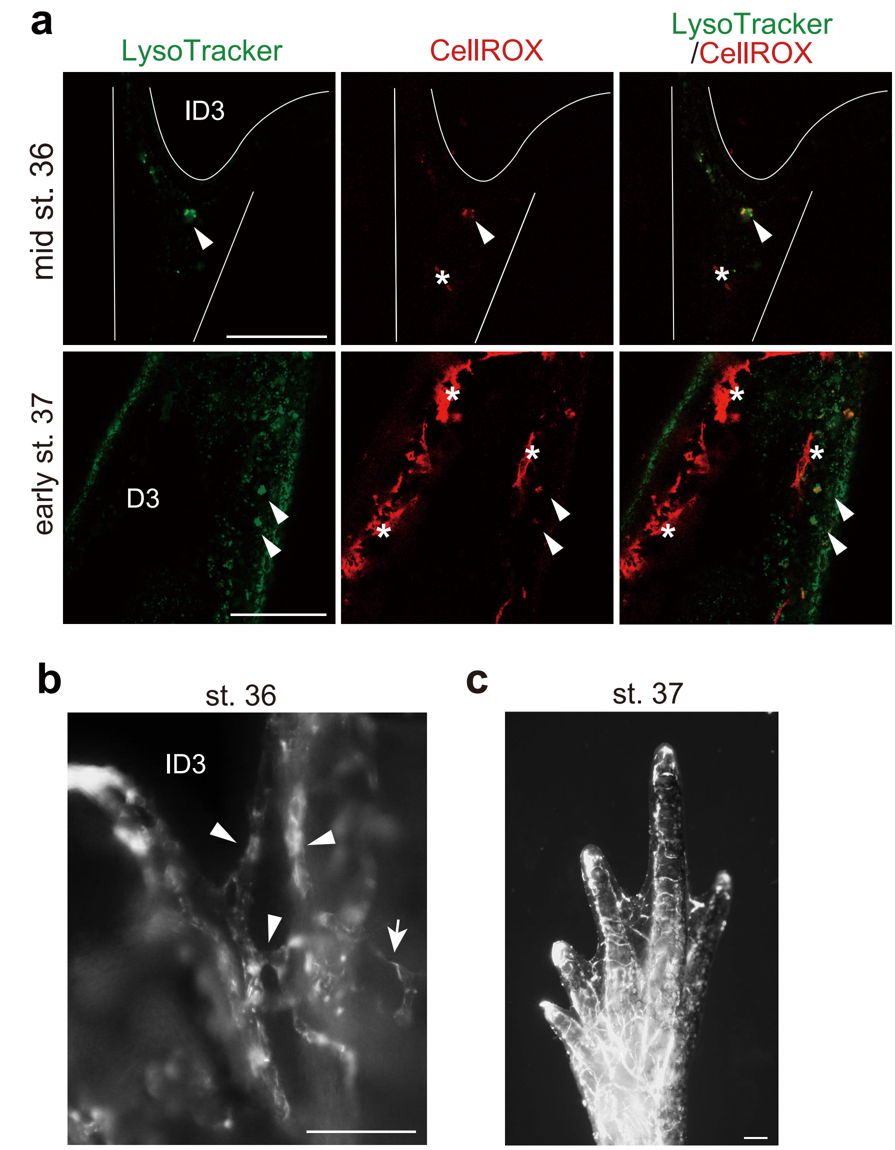

Fig. S5 Cell death, ROS staining and blood vessel pattern

a LysoTracker Green and CellROX staining of *R. pirica* hindlimbs at mid stage 36 and early stage 37. The arrowheads point to both LysoTracker- and CellROX-positive cells. The asterisks indicate pigments, not stained cells. **b** Vasculature (injected with fluorescent ink) of *R. pirica* hindlimbs stage 36. Vessels were observed in interdigital regions, limb margin edges (arrowheads), and surface ectoderm (arrows). **c** Vasculature (injected with fluorescent ink) of *R. pirica* hindlimbs (stage 37). Scale bars, 100 μm. D3: digit 3; ID3: interdigital region 3.
